# Synergistic effect of antibiotics, α-linolenic acid and solvent type against *Staphylococcus aureus* biofilm formation

**DOI:** 10.1007/s43440-024-00669-3

**Published:** 2024-10-28

**Authors:** Karolina Knap, Konrad Kwiecień, Dorota Ochońska, Katarzyna Reczyńska-Kolman, Elżbieta Pamuła, Monika Brzychczy-Włoch

**Affiliations:** 1 Faculty of Materials Science and Ceramics, Department of Biomaterials and Composites, AGH University of Krakow, Al. Mickiewicza 30, Kraków, 30-059 Poland; 2 Faculty of Medicine, Chair of Microbiology, Department of Molecular Medical Microbiology, Jagiellonian University Medical College, ul. Św. Anny 12, Kraków, 31-121 Poland

**Keywords:** Antibiotics, Dimethyl sulfoxide, *Staphylococcus aureus*, Linolenic acid, Biofilm, Quorum sensing inhibition

## Abstract

**Background:**

A promising approach to the treatment of bacterial infections involves inhibiting the quorum sensing (QS) mechanism to prevent the formation and growth of bacterial biofilm. While antibiotics are used to kill remaining bacteria, QS inhibitors (QSIs) allow for antibiotic doses to be reduced. This study focuses on evaluating the synergy between gentamicin sulphate (GEN), tobramycin (TOB), or azithromycin (AZM) with linolenic acid (LNA) against the formation of an early *Staphylococcus aureus* biofilm.

**Methods:**

Minimum biofilm inhibitory concentration (MBIC) was determined using the resazurin reduction assay for all antibiotics and LNA. The reduction of biofilm mass was assessed using the crystal violet (CV) assay. We have also evaluated the effect of dimethyl sulfoxide with TWEEN (DMSO_T) on early biofilm formation. Synergy was determined by metabolic activity assay and fractional biofilm inhibitory concentration (FBIC).

**Results:**

DMSO_T at a concentration of 1% enhanced early biofilm formation, but also decreased the doses of antibiotic needed to reduce the biofilm by up to 8 times. Adding LNA at a concentration of 32 µg/ml or 64 µg/ml allowed up to a 32-fold reduction of antibiotic doses for GEN and TOB and a 4-fold reduction for AZM.

**Conclusions:**

LNA’s use in combination with various antibiotics could reduce their doses and help fight drug-resistant bacteria in the biofilm.

## Introduction

*Staphylococcus aureus* is one of the most common pathogens that cause sepsis, infective endocarditis, skin and soft tissue infections, osteomyelitis, septic arthritis, prosthetic device infections, pulmonary infections, gastroenteritis, meningitis, toxic shock syndrome, urinary tract infections [1]. *S. aureus* occurs in two forms: planktonic and biofilm, the latter being responsible for approximately 80% of all infections [2]. Biofilm formation begins when planktonic forms attach to a surface and begin to colonize it. In the next step, the microcolonies create an extracellular polymeric substance (EPS) [3]. EPS acts as a barrier to antibiotics and protects the bacteria inside the biofilm. EPS is composed of polysaccharides such as polysaccharide intercellular adhesin (PIA) and poly-β(1–6)N-acetylglucosamine (PNAG), external DNA, and proteins [4]. The bacteria hidden in the biofilm are estimated to be more resistant to antibiotics by 10 to 1000 times compared to the planktonic forms [3].

The promising approach to the treatment of bacterial infection focuses on the inhibition of biofilm formation. Quorum sensing (QS) plays an important role in the development, virulence, and antimicrobial resistance of biofilms [5]. The QS mechanism uses small molecules called autoinducers that accumulate in the environment as the density of the bacterial population increases. Bacteria tend to adjust their behaviour to the concentration of autoinducers since this allows estimating the number of population and also altering global patterns of gene expression [6]. One of the well-characterized QS mechanisms in *S. aureus* is the accessory gene regulator (*agr*) [7]. Inhibition of autoinducer production allows for the control of bacterial diseases. Autoinducer production could be interfered with quorum sensing inhibitors (QSIs) [8]. Many QSIs occur as natural compounds present in food, such as α-linolenic acid (LNA) [9, 10]. LNA is an unsaturated fatty acid, which occurs naturally in higher plants and algae [11]. In 1995, Ohta et al. [12] showed the antibacterial activity of LNA against *S. aureus.* They determined the minimal inhibitory concentration (MIC) for the reference strain (*S. aureus* ATCC 25293) and for methicillin-resistant *S. aureus* (MRSA) isolated from a clinical specimen equal to 10 µl/ml and 20 µl/ml, respectively. However, in 2020, Kusumah et al. [13] obtained a MIC of 600 µg/ml for LNA, against *S. aureus* NCTC50581. In addition to antibacterial activity, there are other properties of LNA, such as anti-inflammatory, anticancer, and antioxidant activity, neuroprotection, and regulation of the intestinal microbiota mentioned in the literature [14]. Current theories assume that unsaturated fatty acids may have a different antimicrobial mechanism, which could include disruption of cell-to-cell communication or adenosine triphosphate (ATP) production, alteration of membrane hydrophobicity, blocking enzymes responsible for fatty acid synthesis, causing cellular leakages via increasing membrane poles, and disruption of the electron transport system [15]. In the case of *S. aureus*, the antimicrobial activity of LNA is due to the compound’s ability to disrupt bacterial cell membranes resulting in cell lysis. LNA exerts antimicrobial activity by causing abnormalities on the cell surface or intracellularly. Among other things, carboxylic acids increase membrane permeability as a result of their surfactant properties; in addition, LNA is an inhibitor of oxidative phosphorylation [16–18]. In 2020, Kim et al. [19] showed that LNA reduced the expression of α-hemolysin, a gene associated with the formation of *S. aureus* biofilm.

The QSIs inhibit biofilm formation but do not eliminate or kill bacteria. Therefore, the search for a synergistic effect between QSIs and antibiotics is necessary. One of the most common problems with the testing of QSIs is their relatively poor solubility in water. In these cases, dimethyl sulfoxide (DMSO) is frequently used. The researchers suggested that even low doses of DMSO could influence biofilm formation. The effect of DMSO on biofilm formation depends on specific bacterial strains, their metabolic state, and growth conditions. Therefore, it is always crucial to determine the DMSO effect on bacteria to minimize the risk of biased results, especially since there are studies that omit discussion of such factors [20].

The QSIs prevented or eradicated the biofilm formation, as well as the virulence factors, which allowed for the use of antibiotics at lower doses [21, 22]. This approach is a promising strategy to improve bacterial susceptibility and eliminate multi-drug-resistant bacteria [23].

In our research, we determined the antibacterial activity of gentamicin sulphate (GEN), tobramycin (TOB), azithromycin (AZM), and linolenic acid (LNA) against the planktonic form and the early biofilm of *S. aureus* (ATCC 25923). Also, the influence of LNA in the solvent (DMSO with 2% TWEEN, DMSO_T) on the formation of early *S. aureus* biofilm was evaluated in the presence of antibiotics. Finally, the synergistic effects of antibiotics with LNA on early *S. aureus* biofilm viability were assessed.

## Materials and methods

### Bacterial strains

This study examined *Staphylococcus aureus* (American Type Culture Collection, ATCC 25923). The reduction culture was seeded on Mueller-Hinton agar plates (BioMaxima S.A.) 24 h before the beginning of the experiment and incubated at 37 °C.

### Evaluation of strain susceptibility using the E-test

Single colonies of *S. aureus* were selected from the streak plate and transferred to trypticase soy broth (BioMaxima S.A.) to obtain bacterial suspension at 0.5 McFarland (McF) concentration (1.5·10^8^ CFU/ml). The bacteria were swabbed on Mueller-Hinton agar plates and the E-test with gentamicin (GEN, Merck), tobramycin (TOB, Merck), and azithromycin (AZM, Merck) was placed.

### Solubility of antibiotics and quorum sensing inhibitor

Gentamicin sulphate (GEN; Sigma-Aldrich, cat. no. PHR1077) and tobramycin (TOB; Sigma-Aldrich, cat. no. PHR1079) were dissolved in ultrapure water (Direct-Q3UV, Merck Millipore). Azithromycin (AZM; Sigma-Aldrich, cat. no. PHR1088) was dissolved in 96% ethanol (POCH). All antibiotics were dissolved according to the Clinical and Laboratory Standards Institute standards [24]. The α-linolenic acid (LNA; Sigma-Aldrich, cat. no. L2376-500MG) was dissolved in dimethyl sulfoxide (DMSO, POCH) with 2% TWEEN20 (Sigma-Aldrich) (DMSO_T) [9]. The antibiotics and the QS inhibitor were disinfected with UV light for 20 min.

### Evaluation of Minimum Inhibitory Concentration (MIC)

MIC was evaluated using broth microdilution according to the European Committee on Antimicrobial Susceptibility Testing (EUCAST) guidelines [24]. The individual colonies were selected from the streak plate and transferred to Mueller-Hinton broth (Sigma-Aldrich) to obtain 0.5 McF (1.5·10^8^ CFU/ml) bacterial suspension and diluted 10 times. To glass tubes containing 980 µl Mueller-Hinton broth and 10 µl of dissolved antibiotic/inhibitor in different concentrations, 10 µl of previously obtained bacterial suspension was added (the final concentration was equal to 5·10^5^ CFU/ml). 980 µl broth with 10 µl of solvent (i.e. water for GEN and TOB, ethanol for AZM, or DMSO for LNA) and 10 µl of bacterial suspension were used as a positive control, while the negative control was clear Mueller-Hinton broth. All glass tubes were incubated at 37ºC for 18 h. The MICs were read at the lowest concentration of the agent which completely inhibits the visible growth of bacteria [25].

### Determination of Minimum Bactericidal Concentration (MBC)

After evaluating MIC values, the 100 µl of suspensions were transferred to blood agar plates (BioMaxima S.A.) and spread using sterile baguettes. The plates were incubated at 37ºC for 18 h. The MBC was determined at antibiotic/inhibitor concentrations where the *S. aureus* colonies were not visible.

### Evaluation of Minimum Biofilm Inhibitory Concentration (MBIC)

The single colonies of bacteria were selected from the reduction culture and added to the trypticase in soy broth (BioMaxima S.A.) to obtain a suspension of bacteria at a concentration of 0.5 McF (1.5·10^8^ CFU/ml) or 2 McF (6·10^8^ CFU/ml). The bacteria were seeded in 24-well plates (Costar Corning). To 990 µl of bacterial suspension at 2 McF (6·10^8^ CFU/ml) 10 µl of antibiotic solutions at different concentrations were added. To 980 µl of bacterial suspension at 0.5 McF (1.5·10^8^ CFU/ml) 10 µl of antibiotic solution and 10 µl of DMSO_T were added. For LNA, to 990 µl of bacterial suspension at 0.5 McF (1.5·10^8^ CFU/ml), 10 µl inhibitor solution was added to obtain the final concentration of LNA equal to 256 µg/ml, 128 µg/ml, 64 µg/ml, 32 µg/ml, and 16 µg/ml. The final concentration of DMSO_T was equal to 1% (the final concentration of TWEEN20 was equal to 0.0002%).

The MBIC value was determined using the resazurin reduction metabolic activity test of bacteria within an early biofilm. After 4 h of incubation, the broth with non-attached bacteria was removed and gently washed three times with sterile phosphate-buffered saline (PBS). Then, 1 ml of the soy broth containing 10% v/v AlamarBlue reagent (resazurin sodium salt (Sigma-Aldrich) dissolved in PBS at 0.1 mg/ml) was added to the wells. The plate was incubated at 37ºC for 20 min. Then, 150 µl of soy broth was transferred into a black well plate. Fluorescence was measured at λ_ex_ = 535/25 nm, λ_em_ = 595/35 nm using a microplate reader (SpectraMax^®^ Mini). The percentage of resazurin reduction was calculated (Eq. (1)):

1 $$\eqalign{& Resazurin \,reduction \left( \% \right) \cr & = \,{{{F_x}\, - \,{F_{0\% }}} \over {{F_{100\% }}\, - \,{F_{0\% }}}}\, \cdot \,100\% \cr} $$

where: F_x_ is the fluorescence of the sample, F_0%_ is the fluorescence of soy broth with AlamarBlue reagent without bacteria and F_100%_ is the fluorescence of completely reduced reagent (soy broth with the reagent autoclaved (SMS ASVE) for 15 min at 121 °C).

The bacteria viability was calculated (Eq. (2)):

2 $$\eqalign{& Viability \left( \% \right) \cr & = \,{{Resazurin \,reduction \,of\, sample} \over {The\, mean \,value \,of\, resazurin\, reduction \,for \,control \,sample}} \cr & \cdot \,100\% \cr} $$

The control sample were bacteria within an early biofilm that had not been treated with antibiotics or LNA (only the solvents were added). The MBIC_50_ was the concentration of antibiotic/inhibitor where the viability was lower than 50%, whereas the MBIC_90_ was the concentration where the viability was lower than 90%.

### Biofilm mass reduction

The biofilm mass reduction was evaluated using a crystal violet (CV) assay. After incubation (the bacteria suspension was prepared and incubated under the same conditions as described earlier), the broth with non-attached bacteria was removed. The biofilms were gently washed with sterile PBS three times and allowed to dry at 37ºC. The biofilms were then fixed by adding 1 ml of methanol (POCH) to each well for 20 s. When the biofilms dried, 1 ml of 1% CV (Sigma Aldrich) was added to each well for 20 min. Excess crystal violet staining solution was removed, and the biofilms were washed using sterile water multiple times until the water was visibly clear. The plates with biofilms were placed again at 37ºC and dried overnight.

For biofilm quantification, the plates were placed into a microplate reader (TECAN). The absorbance was measured at 570 nm. The percentage of biofilm mass reduction was calculated (Eq. (3)):

3 $$\eqalign{& Biofilm \,mass \,reduction \left( \% \right) \cr & = \,{{sample } \over {control \,sample}}\, \cdot \,100\% \cr} $$

where: sample is an absorbance of biofilm-treated antibiotic or inhibitor in different concentrations, and the control sample is an absorbance of non-treated biofilm.

### Determination of synergy between antibiotics and QSI

**Fig. 1:**
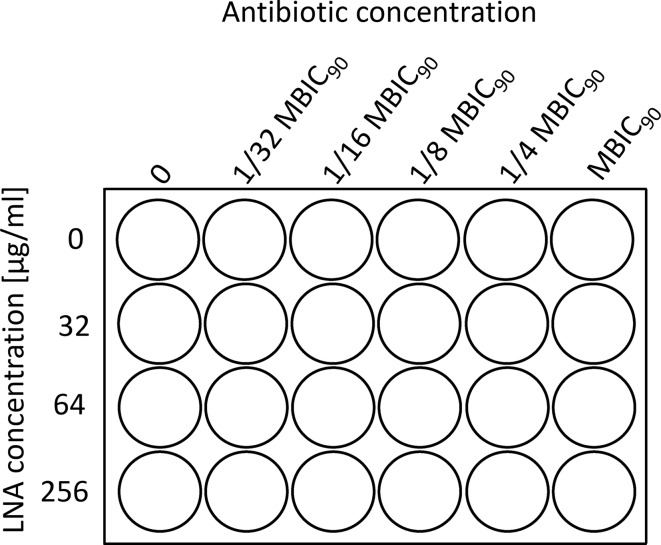
Scheme of the checkerboard. LNA – α-linolenic acid, MBIC_90_ – concentration of antibiotic/inhibitor where the viability was lower than 90%

The synergy of antibiotics and LNA was determined by the checkerboard assay (Fig. 1) as previously described [9] with some modifications. 980 µl of bacterial suspension at 0.5 McF (1.5·10^8^ CFU/ml) was added to each well of a 24-well plate. To the bacterial suspension it was added 10 µl of dissolved antibiotic at final concentrations of 0 µg/ml (first column – control sample), 1/32 MBIC_90_ (second column), 1/16 MBIC_90_ (third column), 1/8 MBIC_90_ (fourth column), 1/4 MBIC_90_ (fifth column), and finally MBIC_90_ (sixth column). Similarly, 10 µl of dissolved LNA was added to the rows at final concentrations of 0 µg/ml (first row – control sample), 32 µg/ml (second row), 64 µg/ml (third row) and 256 µg/ml (fourth row).

The metabolic activity was measured as described earlier. Based on the results, the fractional biofilm inhibitory concentration (FBIC) was calculated (Eq. (4)):

4 $$\eqalign{& FBIC\, = \,FBI{C_A}\, + \,FBI{C_I} \cr & = \,{{MBI{C_{90A\left( I \right)}}} \over {MBI{C_{90A}}}}\, + \,{{MBI{C_{90I\left( A \right)}}} \over {MBI{C_{90I}}}} \cr} $$

Where subscripts A is antibiotic and I an inhibitor (LNA), MBIC_90A(I)_ is an antibiotic MBIC_90_ in the presence of LNA, and MBIC_90I(A)_ is the LNA MBIC_90_ in the presence of an antibiotic. The interaction was defined as follows: FBIC ≤ 0.5, synergism; FBIC ≥ 4, antagonism; FBIC > 0.5 and ≤ 1, additive; and 1 < FBICs < 4, indifference [5, 26].

### Statistics

The statistical analyses of the obtained data were done using a one-way analysis of variance (one-way ANOVA) followed by Tuckey’s post hoc test. The statistical analyses were performed using OriginLab software. The results are presented as mean ± standard deviation (SD). Statistically significant differences were presented in the tables on the graphs with accuracy to 0.0001.

## Results

### Antibacterial activity of antibiotics and linolenic acid against a planktonic form of S. Aureus

The MIC values of antibiotics against *S. aureus* ATCC 25923 were evaluated using two methods: E-tests and according to EUCAST guidelines [24]. On the E-test, the MIC value was equal to 0.125 µg/ml for GEN, 0.38 µg/ml for TOB, and 0.19 µg/ml for AZM, respectively (Table 1). The MICs evaluated according to the ECUAST guidelines were similar to the results of the E-test and were equal to 0.125 µg/ml for GEN and TOB, and 0.5 µg/ml for AZM, respectively. The MBC was determined as the concentration in which the bacteria colonies did not grow on the agar plates after seeding from the post-experimental broth. For AZM and GEN, the MBCs were equal to 1 µg/ml, whereas for TOB it was equal to 0.5 µg/ml. For LNA, the MIC cannot be observed due to the turbidity of the sample due to the presence of water-insoluble LNA, but the MBC was successfully determined and was equal to 1024 µg/ml.

**Table 1:** The results of MIC and MBC evaluated against the planktonic form of *S. Aureus* ATCC 25,923

	MIC evaluated by E-test [µg/ml]	MIC evaluated according to EUCAST guidelines [µg/ml]	MBC [µg/ml]
GEN	0.125	0.125	1
TOB	0.38	0.125	0.5
AZM	0.19	0.5	1
LNA	-	Not visible*	1024

* Not visible due to the turbidity of the sample caused by high LNA concentration

MIC – minimal inhibitory concentration, MBC – minimum bactericidal concentration, GEN – gentamycin sulphate, TOB – tobramycin, AZM – azithromycin, LNA – α-linolenic acid, EUCAST – European Committee on Antimicrobial Susceptibility Testing

### Influence of antibiotics and linolenic acid on *S. Aureus* biofilm formation

**Fig. 2:**
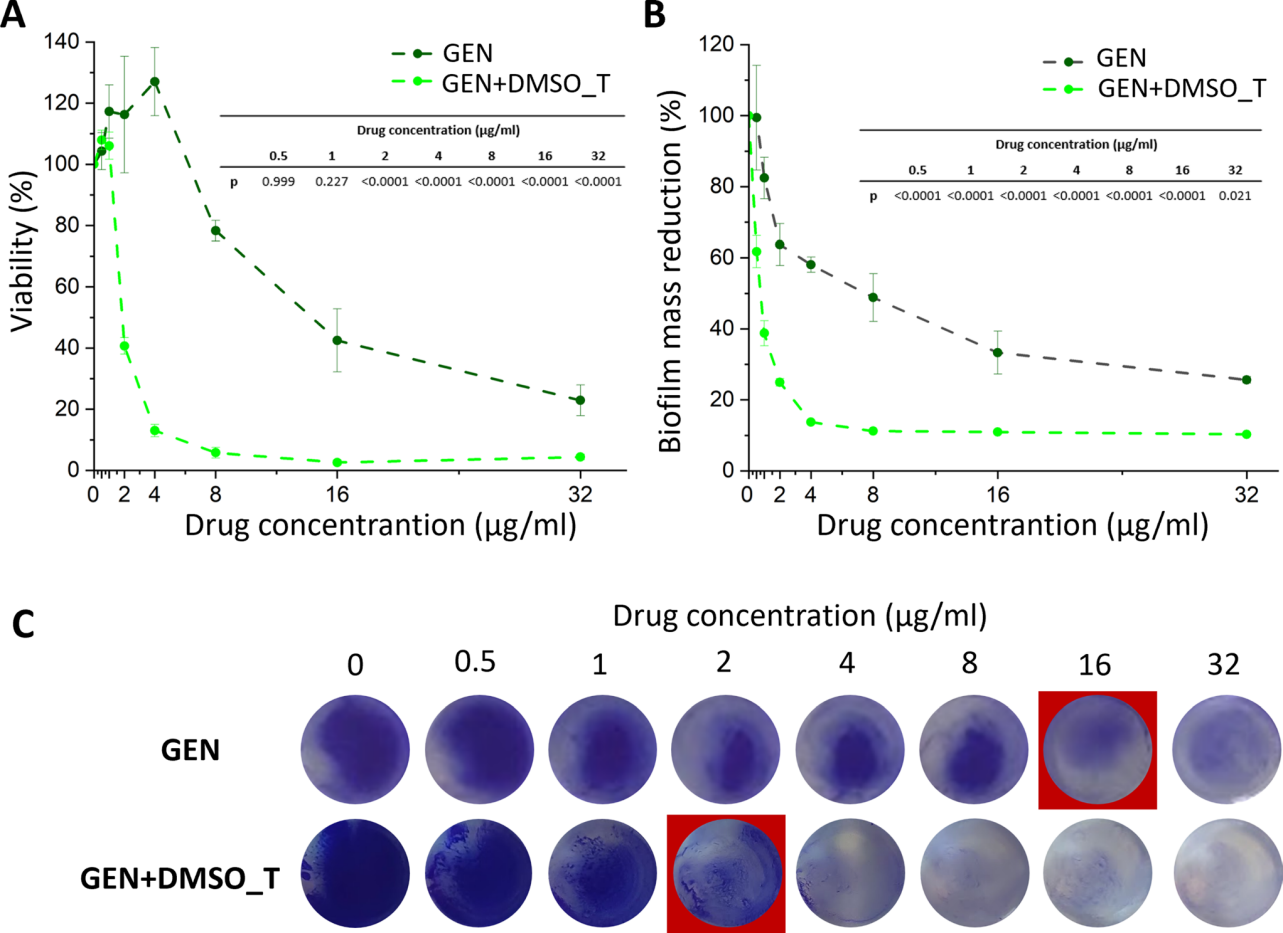
The influence of GEN and GEN + DMSO_T on the viability (determined based on metabolic activity test – AlamarBlue assay) of early biofilm of *S. aureus* ATTC 25923 (**A**), biofilm mass reduction measured by crystal violet absorbance (**B**), and images of crystal violet staining of early biofilm at different GEN and GEN + DMSO_T concentrations (**C**). MBIC_50_ is marked in the red square. The results are presented as mean ± standard deviation (*n* = 3). Statistically significant differences presented in the tables on the graphs with accuracy to 0.0001 (one-way ANOVA followed by Tukey post hoc test). GEN – gentamycin sulphate, GEN + DMSO_T – gentamycin sulphate with the addition of dimethyl sulfoxide and TWEEN

The influence of antibiotics (with or without DMSO_T) and LNA on early biofilm formation of *S. aureus* was evaluated using a metabolic activity test and CV assay. In the case of GEN (Fig. 2), no effects on viability were observed for concentrations equal to or less than 4 µg/ml (F_13,81_=338.56, *p* < 0.001; one-way ANOVA followed by Tukey post hoc test). The viability of the bacteria (Fig. 2A) started to decrease from 8 µg/ml. However, for GEN + DMSO_T the viability started to decline much earlier, i.e. at 2 µg/ml. At 8 µg/ml, the viability was reduced by approximately 94%. A further increase in the concentration of GEN + DMSO_T did not significantly decrease bacterial viability. For GEN, changes in biofilm mass (Fig. 2B) were observed from 1 µg/ml (F_13,25_=92.96, *p* < 0.001; one-way ANOVA followed by Tukey post hoc test). At 8 µg/ml, the mass decreased by approximately 52%. At the same concentration, for GEN + DMSO_T, the mass decreased by approximately 89% and did not change significantly at higher concentrations. Photos of biofilm staining with CV (Fig. 2C) confirm the reduction in biofilm mass.

**Fig. 3:**
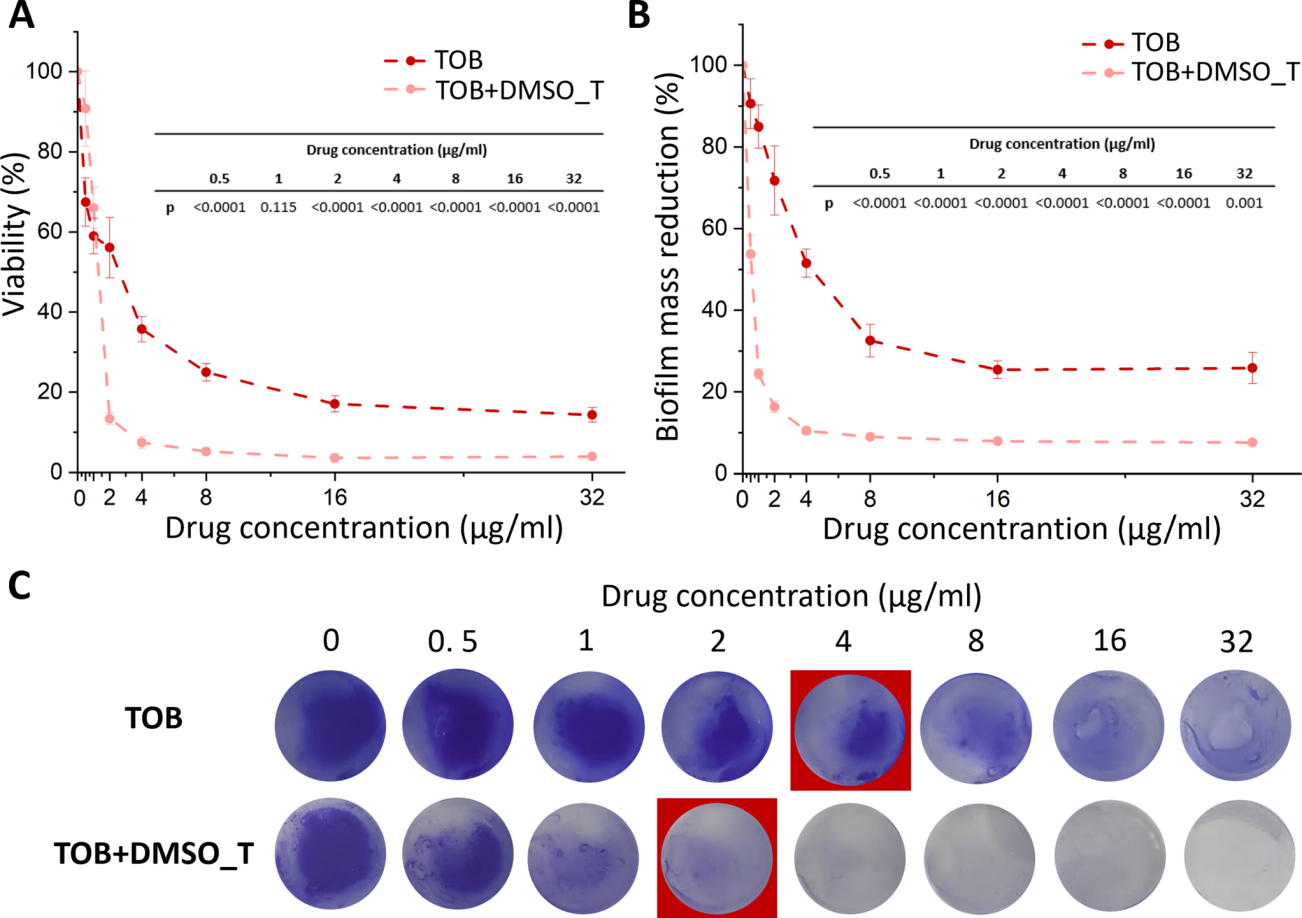
The influence of TOB and TOB + DMSO_T on the viability (determined based on metabolic activity test – AlamarBlue assay) of early biofilm of *S. aureus* ATCC 25923 (**A**), biofilm mass reduction measured by crystal violet absorbance (**B**), and images of crystal violet staining of early biofilm at different TOB and TOB + DMSO_T concentrations (**C**). MBIC_50_ is marked in the red square. The results are presented as mean ± standard deviation (*n* = 3). Statistically significant differences presented in the tables on the graphs with accuracy to 0.0001 (one-way ANOVA followed by Tukey post hoc test). TOB – tobramycin, TOB + DMSO_T – tobramycin with the addition of dimethyl sulfoxide and TWEEN

TOB and TOB + DMSO_T were more effective in the reduction of biofilm in comparison to GEN. The viability of the early biofilm of *S. aureus* (Fig. 3A) was reduced by approximately 42% and 34% of the control already at a concentration of 1 µg/ml of TOB and TOB + DMSO_T, respectively. Increasing TOB concentration caused a subsequent reduction in biofilm viability, whereas, similarly to GEN, the presence of DMSO_T significantly improved the efficiency of the drug (F_13,94_=395.60, *p* < 0.001; one-way ANOVA followed by Tukey post hoc test). For TOB + DMSO_T, the viability was reduced by approximately 87% and 93% at 2 µg/ml and 4 µg/ml, respectively. A further increase in TOB + DMSO_T concentration did not significantly decrease bacterial viability. For TOB alone, the viability at 2 µg/ml and 4 µg/ml was reduced by approximately 44% and 65%, respectively. At the highest concentration tested (i.e. 32 µg/ml), the viability was reduced by approximately 86%. The difference in biofilm mass (Fig. 3B) was also observed (F_13,26_=152.26, *p* < 0.001; one-way ANOVA followed by Tukey post hoc test). For TOB + DMSO_T, at 0.5 µg/ml biofilm mass was reduced by approximately 46%. Increasing the concentration to 4 µg/ml resulted in a further reduction in viability by approximately 90%. However, the higher concentration did not have any significant effect on the biofilm mass. The biofilm mass was reduced by approximately 49% and 75% at 4 µg/ml and 16 µg/ml, respectively. At the highest concentration, no significant changes were observed. The results were compatible with CV staining of the early biofilm (Fig. 3C).

**Fig. 4:**
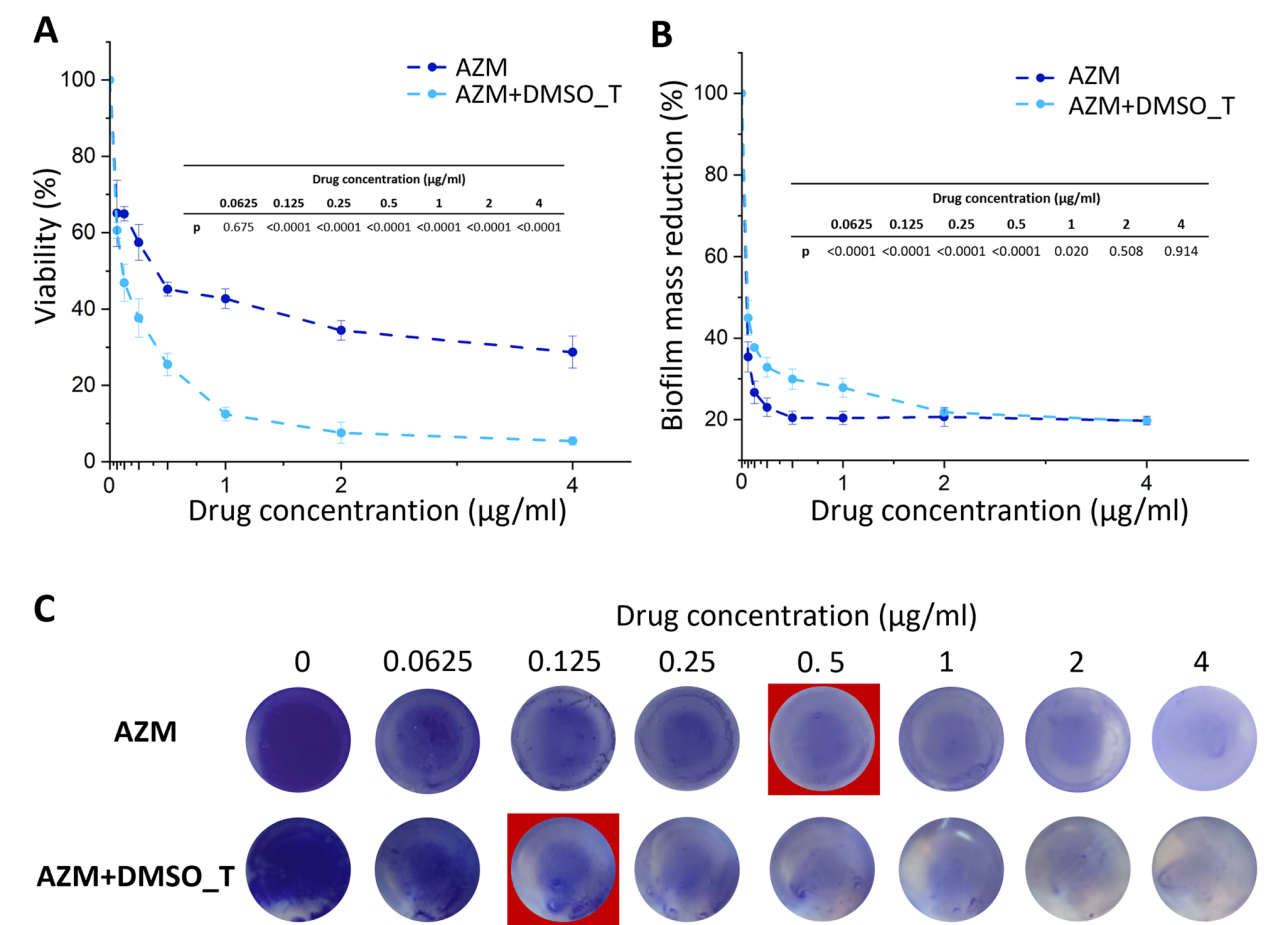
The influence of AZM and AZM + DMSO_T on the viability (determined based on metabolic activity test – AlamarBlue assay) of early biofilm of *S. aureus* ATCC 25923 (**A**), biofilm mass reduction measured by crystal violet absorbance (**B**), and images of crystal violet staining of early biofilm at different AZM and AZM + DMSO_T concentrations (**C**). MBIC_50_ is marked in the red square. The results are presented as mean ± standard deviation (*n* = 3). Statistically significant differences presented in the tables on the graphs with accuracy to 0.0001 (one-way ANOVA followed by Tukey post hoc test). AZM – azithromycin, AZM + DMSO_T – azithromycin with the addition of dimethyl sulfoxide and TWEEN

Among antibiotics tested, AZM was the most effective in the eradication of early *S. aureus* biofilm, thus tested concentrations were decreased to a maximum of 4 µg/ml. The same as in previous antibiotics, AZM in the presence of DMSO_T was more effective. At 2 µg/ml, the viability was reduced by approximately 66% for AZM and 92% for AZM + DMSO_T (Fig. 4A). At the highest concentration, for AZM + DMSO_T, no significant changes were observed (F_13,74_= 169.30, *p* < 0.001; one-way ANOVA followed by Tukey post hoc test). For AZM, the biofilm viability decreased by approximately 72%. Surprisingly, the biofilm mass reduction was very similar for both AZM and AZM + DMSO_T (Fig. 4B). A slight difference was observed at concentrations lower than 2 µg/ml (F_13,28_=38.80, *p* < 0.001; one-way ANOVA followed by Tukey post hoc test). At 2 µg/ml, the biofilm mass reduction was reduced by approximately 80% for AZM and AZM + DMSO_T. At the highest concentration, no significant changes were observed. The results were compatible with CV staining of the biofilm (Fig. 4C).

**Fig. 5:**
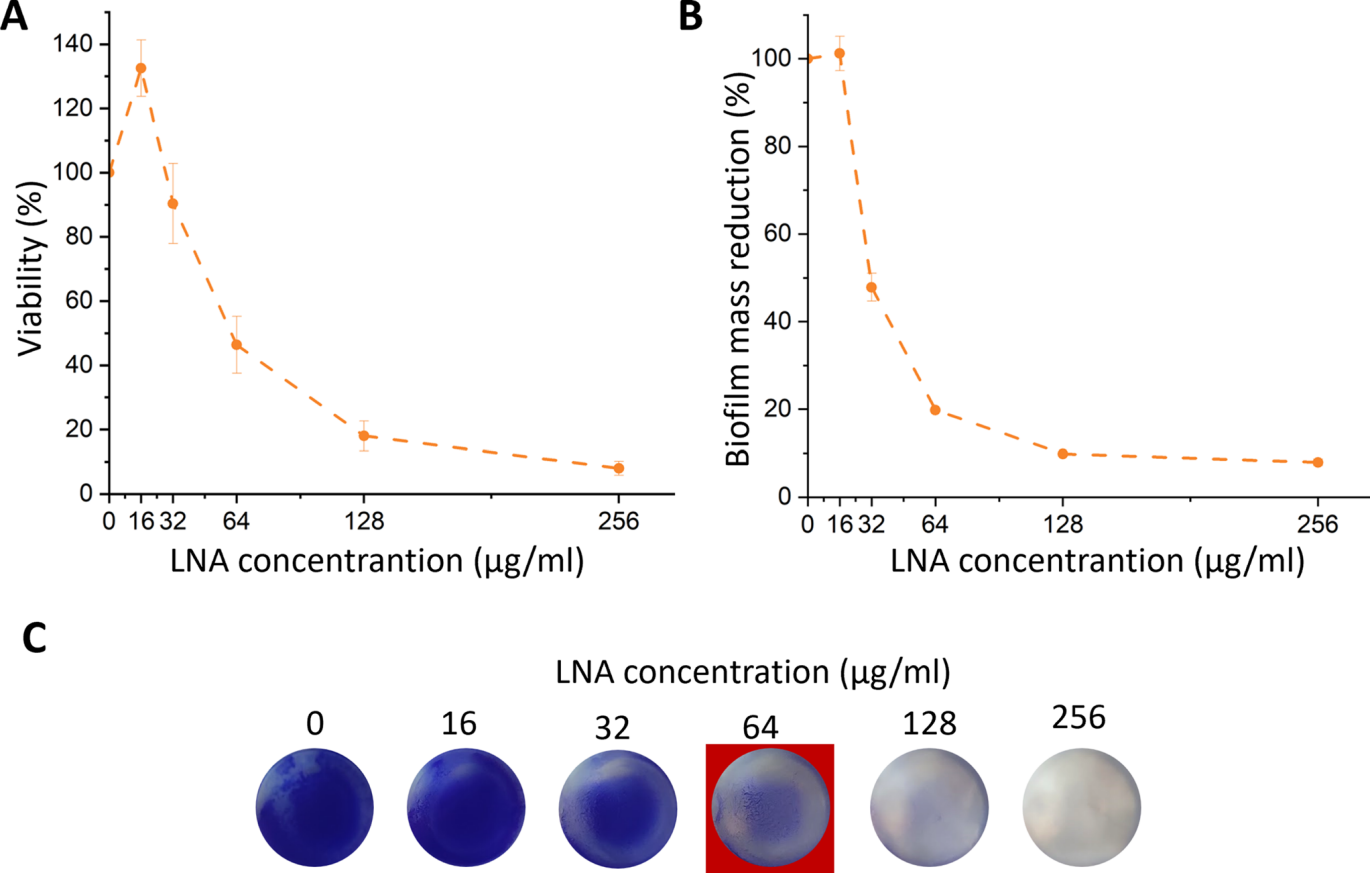
Influence the LNA on the early biofilm of *S. aureus* ATCC 25923 viability (determined based on metabolic activity test – AlamarBlue assay) (**A**), biofilm mass reduction measured by crystal violet absorbance (**B**), and images of crystal violet staining of early biofilm reduction (**C**). The results are presented as mean ± standard deviation (*n* = 3). MBIC_50_ is marked in the red square. LNA – α-linolenic acid

The concentration range tested for LNA was larger compared to antibiotics described previously because LNA was not considered to be such a strong antibacterial agent. The viability of the early biofilm (Fig. 5A) for LNA initially increased at 16 µg/ml, then started to decline. At 32 µg/ml the viability was similar to the control, however at 64 µg/ml was reduced by approximately 54%. At 128 µg/ml and 256 µg/ml, the viability was reduced by approximately 82% and 92%, respectively. The reduction in biofilm mass (Fig. 5B) was significant at concentrations equal to or higher than 32 µg/ml. The results were compatible with CV staining of the biofilm (Fig. 5C).

The addition of DMSO_T to the bacterial suspension decreased the MBIC_50_ for all antibiotics (Table 2). For GEN, the MBIC_50_ was decreased 8 times (from 16 µg/ml to 2 µg/ml), for TOB 2 times (from 4 µg/ml to 2 µg/ml), and for AZM 4 times (from 0.5 µg/ml to 0.125 µg/ml). Additionally, for antibiotics with DMSO_T, MBIC_90_ was successfully determined at all tested concentrations. For GEN it was equal to 8 µg/ml, for TOB 4 µg/ml, and for AZM 2 µg/ml. For LNA, MBIC_50_ was equal to 64 µg/ml and MBIC_90_ 256 µg/ml. On the contrary, MBIC_90_ was not found in samples without DMSO_T, regardless of the antibiotic concentration.

**Table 2:** The results of MBIC_50_ and MBIC_90_ evaluated on early *S. aureus* biofilm

	MBIC_50_ (without DMSO_T) [µg/ml]	MBIC_90_ (without DMSO_T) [µg/ml]	MBIC_50_ (with DMSO_T) [µg/ml]	MBIC_90_ (with DMSO_T) [µg/ml]
GEN	16	Not found at tested concentrations	2	8
TOB	4		2	4
AZM	0.5		0.125	2
LNA	-	-	64	256

MBIC_50_ – concentration of antibiotic/inhibitor where the viability was lower than 50%, MBIC_90_ – concentration of antibiotic/inhibitor where the viability was lower than 90%, DMSO_T – dimethyl sulfoxide with 2% TWEEN20, GEN– gentamycin sulphate, TOB – tobramycin, AZM – azithromycin, LNA – α-linolenic acid

### Synergy between antibiotics and LNA

The synergy between antibiotics and LNA was determined using the AlamarBlue metabolic activity test. The mixtures of antibiotic and LNA at various concentrations were added to the bacterial suspensions for 4 h. After 4 h of incubation, the MBIC_90_ was determined and used for the calculation of FBICs values (sum of dividing the MBIC_90_ antibiotic in the presence of LNA by the MBIC_90_ antibiotic and the MBIC_90_ LNA in the presence of the antibiotic by the MBIC_90_ LNA).

**Fig. 6:**
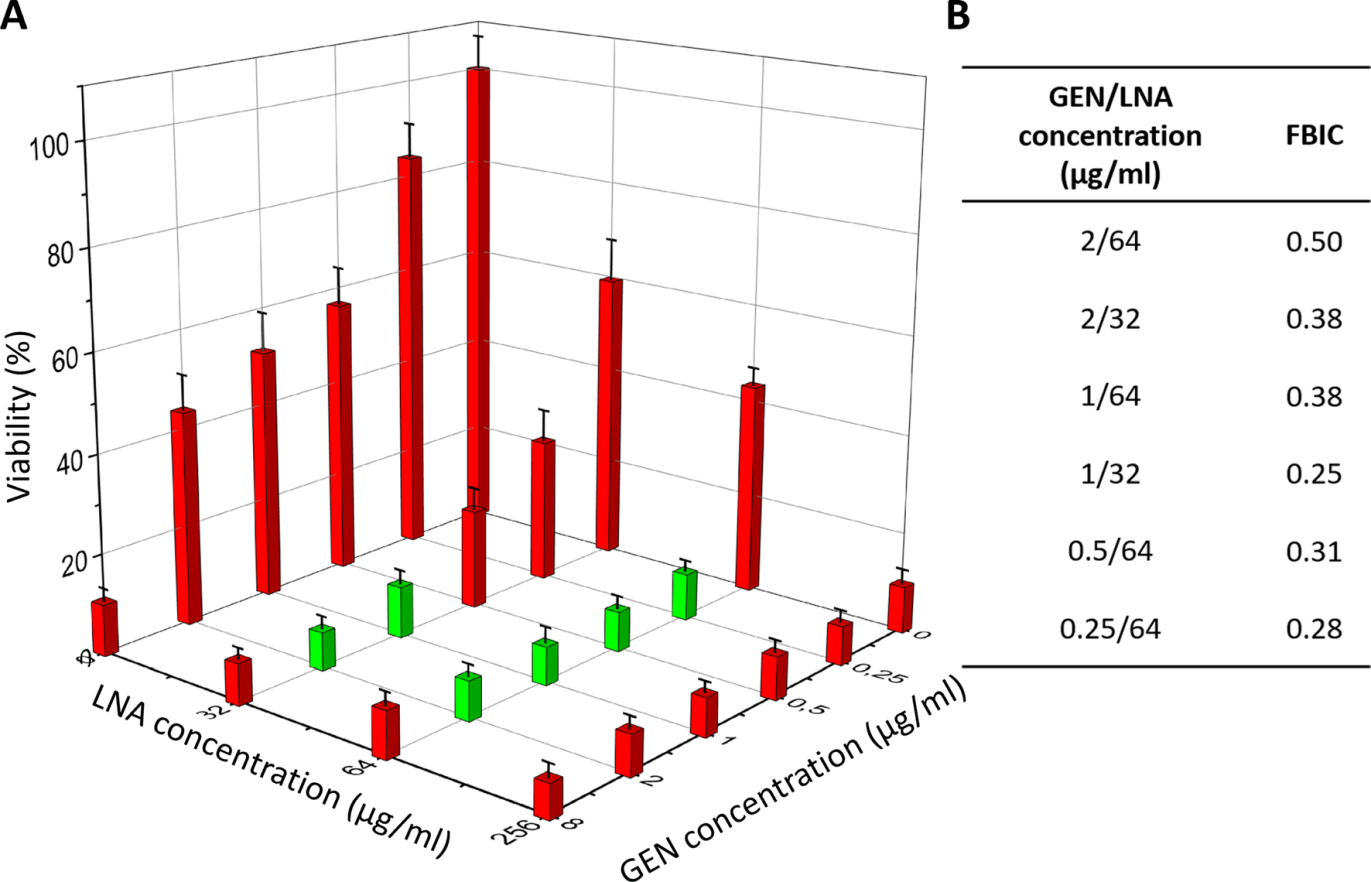
Synergy determination (green bars) between LNA and GEN against biofilm of *S. aureus* ATCC 25923 viability (determined based on metabolic activity test – AlamarBlue assay) (**A**) and FBIC values calculated for synergistic combinations based on MBIC_90_ (**B**). The results are presented as mean ± standard deviation (*n* = 3). LNA – α-linolenic acid, GEN – gentamycin sulphate, FBIC – fractional biofilm inhibitory concentration, MBIC_90_ – concentration of antibiotic/inhibitor where the viability was lower than 90%

For GEN and LNA, the synergy was found in 6 combinations (marked with green bars in Fig. 6A and the calculated FBCIs values in Fig. 6B). The synergy was observed for GEN at concentrations ranging from 2 µg/ml to 0.25 µg/ml in the presence of 32 µg/ml or 64 µg/ml LNA. In these cases, to obtain the same effectiveness as GEN alone at a dose of 8 µg/ml, it was possible to reduce the dose of GEN from 4 to 32 times in the presence of LNA. The early biofilm viability for MBIC_90_ for LNA and GEN alone (9.5% ± 3.0% and 10.4% ± 2.6%, respectively) was similar for synergistic combinations.

**Fig. 7:**
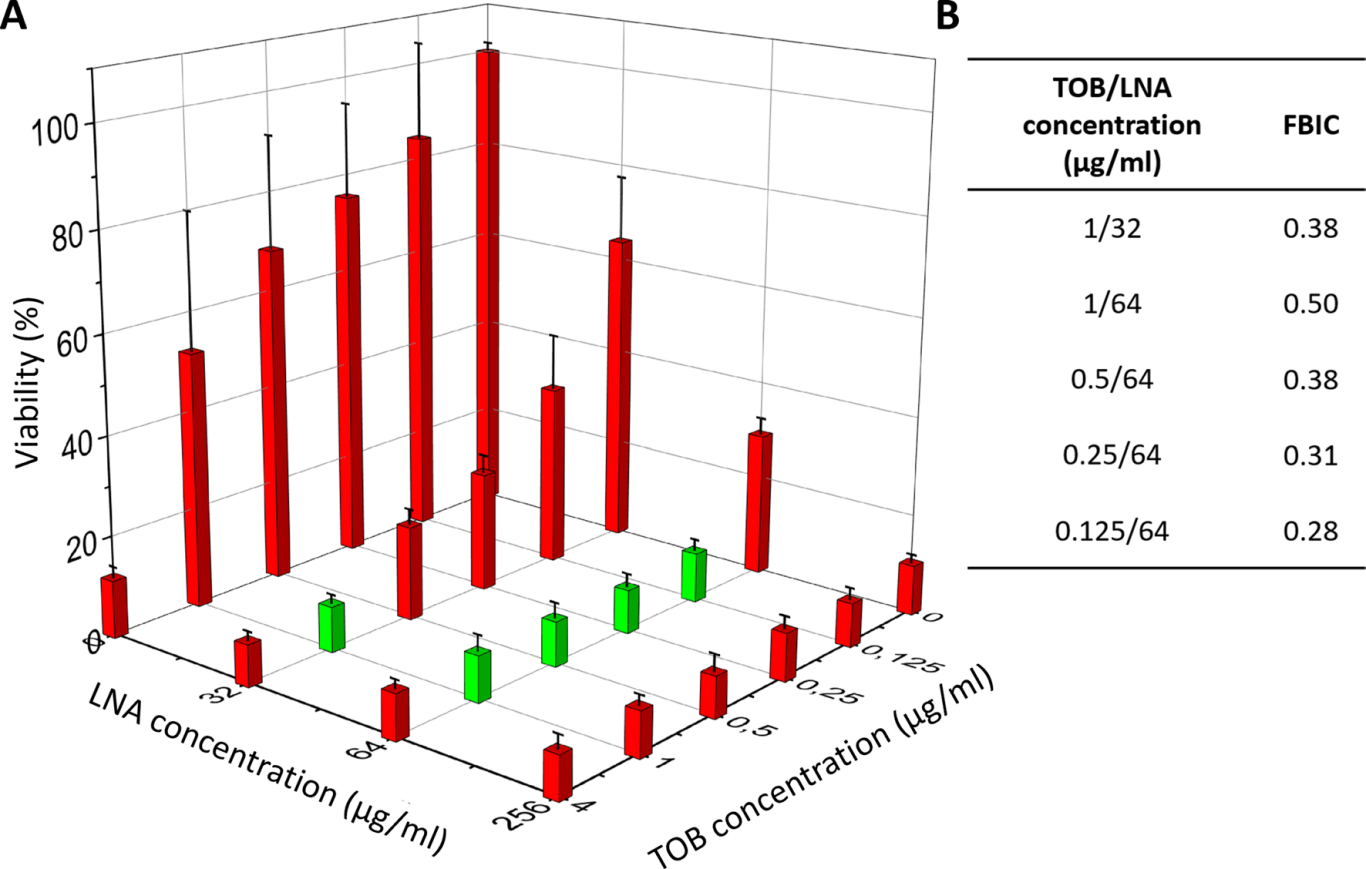
Synergy determination (green bars) between LNA and TOB against biofilm of *S. aureus*: ATCC 25923 viability (determined based on metabolic activity test – AlamarBlue assay) (**A**) and FBIC values calculated for synergistic combinations based on MBIC_90_ (**B**). The results are presented as mean ± standard deviation (*n* = 3). LNA – α-linolenic acid, TOB – tobramycin, FBIC – fractional biofilm inhibitory concentration, MBIC_90_ – concentration of antibiotic/inhibitor where the viability was lower than 90%

For TOB and LNA the synergy was found at five tested concentrations (marked with green bars in Fig. 7A and the calculated FBCIs values in Fig. 7B). The synergy was observed at TOB concentrations ranging from 1 µg/ml to 0.125 µg/ml with the presence of 32 µg/ml or 64 µg/ml LNA. In this case, it was possible to decrease the TOB dose from 4 to 32 times, obtaining the same results as using TOB alone at a concentration equal to 8 µg/ml. The biofilm viability for MBIC_90_ for LNA and TOB alone (10.2% ± 1.7% and 11.6% ± 2.1%, respectively) was similar for synergistic combinations.

**Fig. 8:**
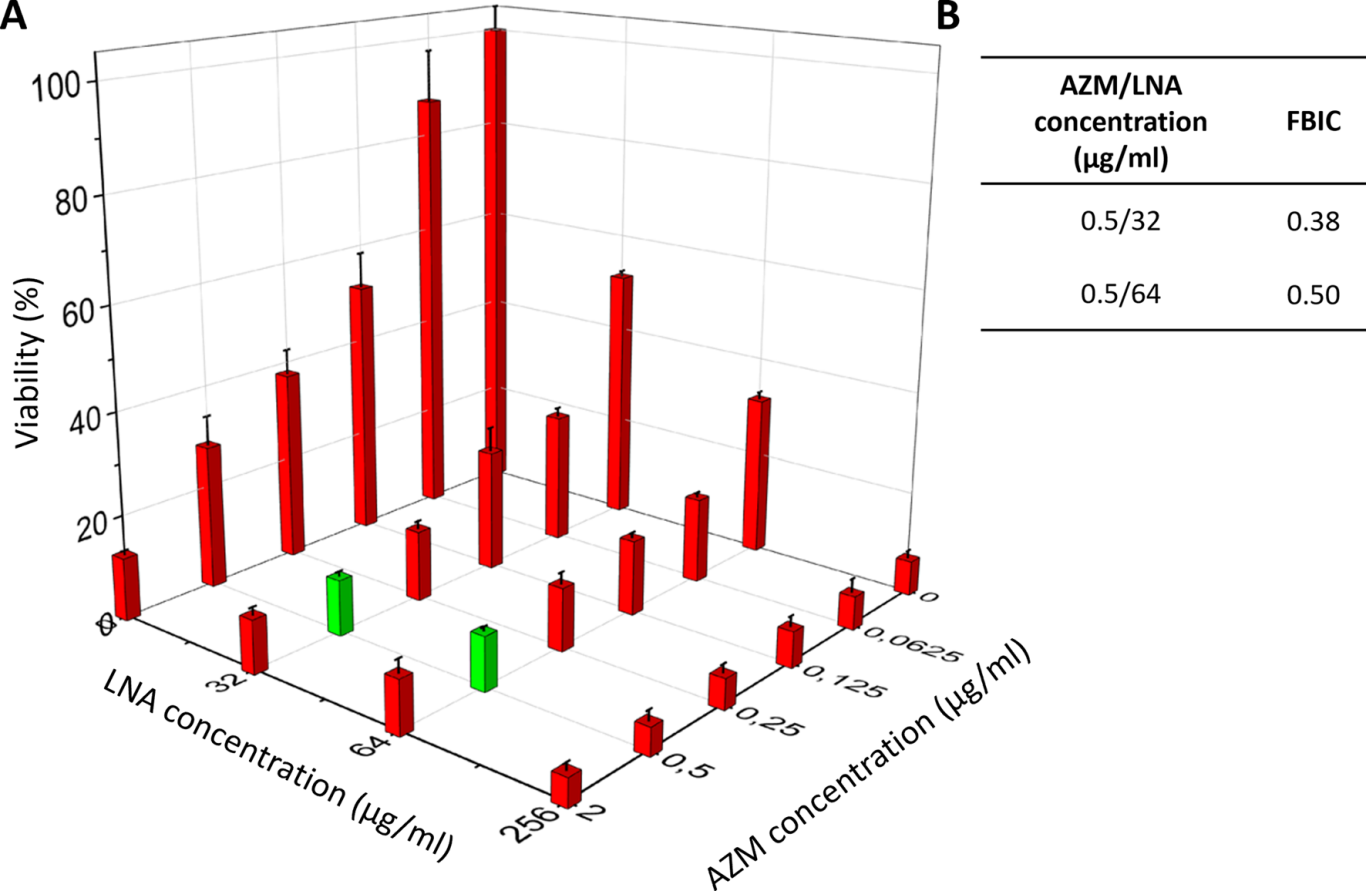
Synergy determination (green bars) between LNA and AZM against biofilm of *S. aureus* ATCC 25923 viability (determined based on metabolic activity test – AlamarBlue assay) (**A**) and FBIC values calculated for synergistic combinations based on MBIC_90_ (**B**). The results are presented as mean ± standard deviation (*n* = 3). LNA – α-linolenic acid, AZM – azithromycin, FBIC – fractional biofilm inhibitory concentration, MBIC_90_ – concentration of antibiotic/inhibitor where the viability was lower than 90%

For AZM and LNA the synergy was found in only two combinations (marked with green bars in Fig. 8A and the calculated FBCIs values in Fig. 8B). The synergy was observed at an AZM concentration equal to 0.5 µg/ml in the presence of 32 µg/ml or 64 µg/ml LNA. In this case, it was possible to decrease the dose of AZM 4 times, obtaining the same results as using AZM alone at a concentration equal to 2 µg/ml. The biofilm viability for MBIC_90_ for LNA and AZM alone (6.8% ± 1.5% and 12.3% ± 0.80, respectively) was similar for synergistic combinations.

## Discussion

In our study, we determined the synergy between LNA and different antibiotics to eradicate the early *S. aureus* biofilm. The addition of 32 µg/ml or 64 µg/ml LNA to antibiotics allowed the antibiotic doses to be reduced even 32 times. Additionally, we observed that DMSO_T enhanced the early biofilm formation. Moreover, the addition of DMSO_T decreased the antibiotic doses required to eradicate the biofilm.

In the beginning, the influence of LNA on the planktonic form and biofilm formation of *S. aureus* was evaluated. The LNA killed the bacteria in the planktonic form at 1024 µg/ml, and also had an impact on biofilm formation. MBIC_90_ was determined and was equal to 256 µg/ml. The previously determined MIC values of LNA against *S. aureus* were 1560 µg/ml and 600 µg/ml reported by McGaw et al. [27] and Kusumah et al. [13], respectively. The differences between results would be associated with the use of different solvents. In our study, we used DMSO_T, whereas Kusumah et al. [13] dissolved the LNA in ethanol. Lee et al. [28] previously confirmed the antibiofilm properties of LNA against *S. aureus*. Cepas and Gutiérrez-del-Río et al. [29] evaluated MBIC_50_ against *S. aureus* S54F9 for LNA and found that it was equal to 64 µg/ml. In this study, we determined the same value of MBIC_50_ against *S. aureus*.

In our study, we dissolved LNA in DMSO_T because LNA is insoluble in water. We evaluated the influence of the solvent on biofilm formation to eliminate the risk of bias. The significant impact of DMSO on the formation of, for example, *Escherichia coli* biofilm was confirmed by Lim et al. [30]. In their research, the biofilm formation increased by more than 100% in the presence of 4% DMSO. Hassan et al. [31] confirmed that in Gram-negative bacteria (*E. coli*) the presence of an outer membrane results in reduced permeability to DMSO. The cell wall of Gram-positive bacteria (*S. aureus*) lacks an outer membrane, which facilitates the penetration of DMSO into the interior of the Gram-positive bacterial cell. However, to the best of our knowledge, it has not been investigated so far. Therefore, we evaluated the biofilm viability in the presence of antibiotics and DMSO_T. Depending on the concentrations and bacteria strains, DMSO can affect bacteria in different ways. At high concentrations (> 10%), DMSO has strong antimicrobial and antibiofilm properties, while at lower concentrations (< 10%) DMSO can stimulate biofilm formation [20].

The effect of TWEEN20 on the formation of *E. coli* biofilm was described by Wu et al. [32]. A slight decrease in biofilm formation was observed in the presence of TWEEN20 at concentrations equal to or below 0.001%. However, at higher concentrations, the biofilm increased. Nguyen et al. [33] demonstrated only weak disruptive effects of TWEEN20 on the biofilm of *P. aeruginosa* at 1% wt. In our study, we used DMSO_T at a concentration of 1% (the concentration of TWEEN20 was equal to 0.0002%) and, as expected, observed its stimulative effect on biofilm formation. A similar biofilm was observed at an initial bacterial concentration of 2 McF (6·10^8^ CFU/ml) without DMSO_T and 0.5 McF (1.5·10^8^ CFU/ml) with DMSO_T. However, adding the antibiotic to the bacterial suspension with the same DMSO_T ratio reduced the MBIC values. This suggests that DMSO somehow increases the potency of antibiotics, possibly by increasing permeability through the bacterial membrane [34]. However, the mechanism of action of DMSO on the biofilm is not well described. DMSO may disrupt polymer chain aggregation in polysaccharides (i.e., the main component of bacterial EPS matrices), chemically reduce the critical components of QS pathways, inhibit functional protein linkages, and alter the electrostatic charge, solubility, and interactions between polysaccharides and proteins to weaken the overall adhesion forces between the biofilm and the surface [20].

*S. aureus* infections are commonly treated with aminoglycosides. Their mechanism of action is based on the ability to bind to bacterial ribosomes, leading to inhibition of protein synthesis by promoting mistranslation and eliminating proofreading [35]. In our study, we used two aminoglycosides: GEN and TOB. The MIC values for both antibiotics were similar to the results of the E-test. However, the concentrations required to inhibit biofilm formation were higher. Specifically, MBIC_50_ was 128 times higher than the MIC for GEN and 32 times higher than the MIC for TOB. Higher concentrations are required to eliminate the biofilm, which is associated with a higher bacterial density and EPS matrix formation [36]. Without the addition of DMSO_T, the antibiotic efficacy against *S. aureus* biofilm was different: MBIC_50_ was 4 times higher for GEN than for TOB. After the addition of DMSO_T, MBIC_50_ was the same for both antibiotics; however, MBIC_90_ was still two times higher for GEN. For antibiotics without the addition of DMSO_T, MBIC_90_ was higher than the concentrations tested. Guo et al. [37] evaluated the effect of DMSO on the MIC of ciprofloxacin against *Pseudomonas aeruginosa*. The MIC was reduced from 0.4 µg/ml (without the addition of DMSO) to 0.2 µg/ml in the presence of DMSO at concentrations of 1% and 2%.

Another group of antibiotics for the eradication *S. aureus* infection are macrolides, including AZM. Their mechanism of action is different, but they also inhibit protein synthesis. Disruption of bacterial protein synthesis involves binding to the 50 S ribosomal subunit and, ultimately, inhibiting microbial growth [38]. The difference between aminoglycosides and AZM is observed in the reduction of the biofilm mass. In the case of aminoglycosides, the biofilm mass decreased faster for samples with the addition of DMSO_T. For AZM and AZM + DMSO_T, the biofilm mass reduction was very similar. This may be related to the mechanism of action, because aminoglycosides can kill bacteria, while macrolides, such as AZM, inhibit their growth only without affecting existing microorganisms.

The synergistic effect of LNA and antibiotics was evaluated by calculating FBIC as previously described [26, 39–42]. FBIC is the sum of dividing the antibiotic MBIC_90_ in the presence of LNA by the antibiotic MBIC_90_ and the LNA MBIC_90_ in the presence of the antibiotic by the LNA MBIC_90_. In our work, we determined MBIC_90_ using a metabolic activity test similar to Maset et al. [43] or Mahmoudabadi et al. [44]. However, in some works, the authors used a CV assay to determine MBIC_90_ [41, 42, 45]. In our opinion, it is better to use a metabolic activity test to evaluate MBIC_90_ because CV staining cannot show whether the bacteria inside the biofilm are alive or not. In 2017, Chanda et al. [9] reported the synergistic effect of LNA and TOB against *P. aeruginosa*. In that study, synergy was found at the concentration of LNA and TOB of 390 µg/ml and 78 µg/ml, respectively. This allowed a 4-fold reduction in antibiotic dose. In our study, we used lower concentrations of LNA (32 and 64 µg/ml) and achieved a 32-fold reduction in TOB dose (in combination with 64 µg/ml LNA and 0.125 µg/ml TOB). A similar reduction was observed for the combination of LNA and GEN. The synergistic antimicrobial and antibiofilm activity of LNA with aminoglycoside antibiotics (GEN and TOB) results in the interaction of LNA with the cell membrane, increasing its permeability, and facilitating the transport of the aminoglycoside antibiotic in biofilm cells. Furthermore, the antibiofilm effect of the aminoglycoside is due to facilitated penetration of the biofilm matrix due to the action of LNA as a surfactant [17]. For AZM and LNA, the dose reduction was 4 times. The synergistic antimicrobial effect of LNA on macrolide antibiotic is due to interference with the MsrA efflux pump in *S. aureus* [17, 46]. The dose of LNA is safe, as the recommendations of the European Commission state that the amount of fatty acid in infant formulas requires that LNA should be included at a level of at least 50–100 mg/100 kcal, leading to the consumption of its doses much higher than those used in our study [47].

## Conclusion

QSI, such as LNA, has the potential to prevent *S. aureus* biofilm formation. LNA also has an effect on the planktonic form, but only at high concentrations. The ability of *S. aureus* to form a biofilm depends on the type of solvent used to dissolve active pharmaceutical ingredients. In our study, we observed that DMSO_T increased the ability to form the biofilm. Furthermore, low concentrations of DMSO_T affect the effectiveness of antibiotics by reducing the dose necessary to limit the metabolic activity of the biofilm. Combining antibiotics with LNA leads to a further reduction in dose as a result of the synergistic effect of antibiotics and LNA. This approach shows promise in the treatment of *S. aureus* infections and may help combat antibiotic resistance in biofilms. However, to confirm the efficiency of this synergy in the treatment of *S. aureus* infections, it is necessary to test this combination on different reference and clinical bacterial strains.

## Data Availability

Data are available from the corresponding authors upon request.

## Abbreviations

ATP: adenosine triphosphate
AZM: azithromycin
CV: crystal violet
DMSO: dimethyl sulfoxide
DMSO_T: dimethyl sulfoxide with 2% TWEEN20
EPS: extracellular polymeric substance
EUCAST: European Committee on Antimicrobial Susceptibility Testing
FBIC: fractional biofilm inhibitory concentration
GEN: gentamycin sulphate
LNA: α-linolenic acid
MBC: Minimum Bactericidal Concentration
MBIC: Minimum Biofilm Inhibitory Concentration
McF: McFarland
MIC: Minimum Inhibitory Concentration
MRSA: methicillin-resistant *Staphylococcus aureus*
PIA: polysaccharide intercellular adhesin
PNAG: poly-β(1–6)N-acetylglucosamine
QS: quorum sensing
QSI: quorum sensing inhibitor
TOB: tobramycin
